# A Clinical Study of Ninjin'yoeito With Regard to Frailty

**DOI:** 10.3389/fnut.2018.00073

**Published:** 2018-09-24

**Authors:** Naoya Sakisaka, Kazuo Mitani, Sadahiro Sempuku, Tamaki Imai, Yoshinori Takemoto, Hiroaki Shimomura, Takahisa Ushiroyama

**Affiliations:** ^1^Sakisaka Clinic, Osaka, Japan; ^2^Mitani Family Clinic, Osaka, Japan; ^3^Sempuku Clinic, Osaka, Japan; ^4^Imai Clinic, Osaka, Japan; ^5^Takemoto Clinic, Nara, Japan; ^6^Shimomura Clinic of Internal Medicine, Osaka, Japan; ^7^Osaka Medical College Health Science Clinic, Osaka, Japan

**Keywords:** ninjin'yoeito, frailty, sarcopenia, kampo, elder

## Abstract

Frailty in older people is strongly associated with poor nutrition, which is particularly important in the present-day superaging society. This study initially investigated a number of cases of frailty where there was a speedy recovery after administration of a dual deficiency of qi and blood preparation, ninjin'yoeito (NYT), formulated for frail patients who suffer from kiketuryokyo status. Based on these observations, a more extensive investigation involving a greater number of cases was completed. The findings of the effects of NYT on frailty are reported here.

## Introduction

As Japan's population rapidly ages, the problems of “poor nutrition” and “nutritional deficiency” in a superaging society have grown in importance. Frailty in elderly people is strongly associated with poor nutrition, and it can cause sarcopenia, which manifests in a decrease in muscle mass (1). To resolve such issues, the Ministry of Health, Labor, and Welfare announced that starting in 2018 it will implement nationwide “Frailty” countermeasures (2).

Those who work in health care for the elderly understand that people shift between frail and healthy conditions, so appropriate interventions should be made as necessary. When treating frailty through nutritional therapy, the active intake of protein, vitamin D, vitamin E, vitamin C, and so on has been recommended (3). As for exercise therapy, strength training at a slightly intense level, two to three times a week, in addition to aerobic exercise, such as walking, is recommended (4). However, there has been little research on the use of medication to treat frailty and sarcopenia.

In a previous study, the author administered a dual deficiency of qi and blood preparation, NYT, to patients affected by frailty and who were suffering from *kiketuryokyo*, and found that in a number of cases, the patients recovered quickly from a state of frailty to a healthy condition (5). On the basis of the findings of the previous study, we carried out a cohort study that examined the effects of NYT on frailty. These findings are reported here.

## Study subjects

The subjects were chosen from six medical facilities that participated in this research study from April 2016 to March 2018. They were elderly patients who were over the age of 65 and could walk without assistance, and who had at least one of the following symptoms: decreased strength after illness, malaise, hypophagia, night sweats, cold hands and feet, or anemia. The exclusion criteria included the following: (1) patients who had taken *Kampo* (i.e., Chinese medicine) or crude drugs within the 2 weeks before the commencement of the study; (2) obese patients with a body mass index (BMI) of over 25; and (3) patients whose physician in charge deemed their involvement in the study to be unsuitable. Furthermore, a group of patients whose background matched the study's criteria was set up as the control group.

## Method

*NYT group*: Kracie Ninjin'yoeito Granule Extract, which contains 7.5 g of ninjin'yoeito, was given over two to three administrations throughout the day, delivered orally before or during meals. Right and left grip strength, weight, BMI, muscle mass, body fat percentage, lean body mass, muscle quality score, estimated bone mass, body age, and thigh circumference were measured at 0, 8, 16, and 24 weeks. All variables, except grip strength and thigh circumference, were measured using a body composition meter RD-903 or RD-501 (Tanita Corporation, Tokyo, Japan). Statcel 3 software (OMS Publishing Inc., Saitama, Japan) was used as the statistical analysis software.

*Control group*: No placebos were administered as the group was observed and continued with their conventional treatment. The same items as the NYT group were measured. The envelope method was used as a method of grouping.

This study has been screened by an ethical committee that included the presence of a lawyer and third parties (Medical Corporation Sakisaka Clinic Ethical Committee, approval number: 160201).

## Results

NYT group: 64 patients (male/female = 18/46), average age 78.5 ± 6.5 years.

Control group: 49 patients (male/female = 17/32), average age 76.0 ± 6.3 years (Table 1).

**Table 1:** Patient background.

	**Ninjin'yoeito Group (*n* = 64)**	**Control group (*n* = 49)**	***P*-value**
Age, years	78.5 ± 6.5	76.0 ± 6.3	^*^
Sex (Male/Female)	18/46	17/32	n.s.
Height, cm	153.1 ± 9.1	156.3 ± 8.2	n.s.
Right-hand grip strength, kg	18.0 ± 5.7	22.1 ± 7.2	^**^
Left-hand grip strength, kg	16.9 ± 5.9	21.5 ± 7.5	^**^
Weight, kg	50.7 ± 9.7	53.7 ± 8.8	n.s.
BMI, kg/m^2^	21.5 ± 2.9	22.0 ± 2.8	n.s.
Body fat percentage, %	26.8 ± 9.4	25.3 ± 9.2	n.s.
Muscle mass, kg	34.1 ± 7.1	37.9 ± 6.5	^*^
Muscle quality score, points	41.8 ± 17.0	42.6 ± 16.0	n.s.
Estimated bone mass, kg	2.0 ± 0.5	2.2 ± 0.4	^*^
Body age, years	69.7 ± 7.5	66.1 ± 8.1	^*^
Thigh circumference, cm	42.5 ± 5.7	42.1 ± 4.9	n.s.
Lean body mass, kg	36.8 ± 6.4	40.2 ± 7.1	^*^

The comparisons between groups for variables, other than sex, were carried out using Welch's t-test. The comparison between groups for sex was carried out using the chi-square test. Both sides were tested at p < 0.05 (^*^), p < 0.01 (^**^).

Because the missing value was 8 weeks' data and 16 weeks' data, it was compared at 0 and 24 weeks.

### Grip strength

**Figure 1:**
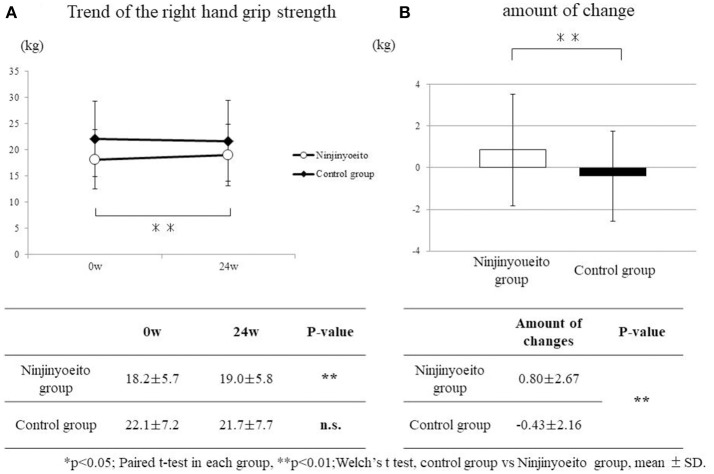
Change in right-hand grip strength between both groups. The grip strength of the groups was compared using the paired *t*-test. The amount of change of each group was compared using Welch's *t*-test. Both sides were tested at *p* < 0.05 (*), *p* < 0.01 (**). Ninjin'yoeito group: 61 cases; control group: 49 cases. A significant improvement was seen in the ninjin'yoeito group. There were no changes in the control group. With regard to the amount of change before and after administration, when both groups were compared, the ninjin'yoeito group showed significant improvement.

For the right-hand grip strength, the NYT group had significantly improved (*p* < 0.01). There was no change in the control group. With regard to the amount of change before and after administration, when the two groups were compared, a significant improvement was seen in the NYT group (*p* < 0.01) (Figure 1).

**Figure 2:**
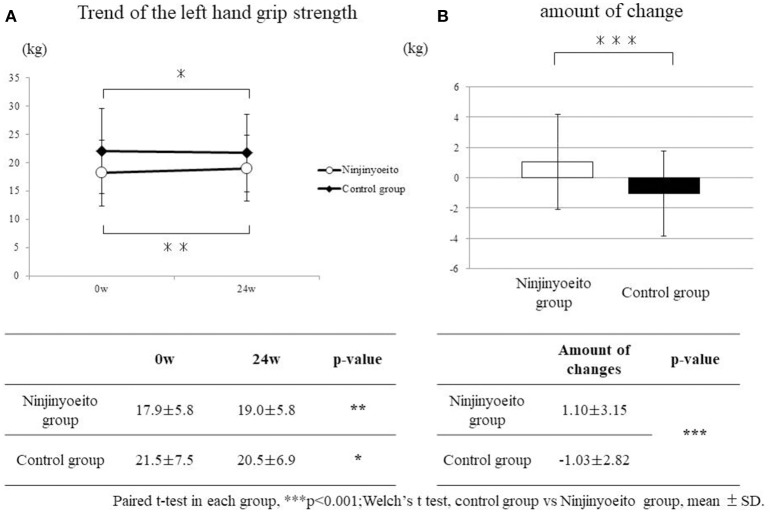
Change in left-hand grip strength between both groups. The grip strength of the groups was compared using the paired t-test. The amount of change of each group was compared using Welch's *t*-test. Both sides were tested at *p* < 0.05 (*), *p* < 0.01 (**), *p* < 0.001 (***). Ninjin'yoeito group: 61 cases; control group: 49 cases. A significant improvement was seen in the ninjin'yoeito group. Left-hand grip strength had significantly deteriorated in the control group. With regard to the amount of change before and after administration, when both groups were compared, the ninjin'yoeito group showed significant improvement.

For the left-hand grip strength, the NYT group had significantly improved (*p* < 0.01), whereas the control group had significantly deteriorated (*p* < 0.05). With regard to the amount of change before and after administration, when the two groups were compared, a significant improvement was seen in the NYT group (*p* < 0.001) (Figure 2).

### Weight and BMI

For weight and BMI, there were no changes in the NYT group or the control group (data not shown).

### Muscle mass

There was no change in the NYT group, but in the control group, muscle mass had significantly deteriorated (*p* < 0.05). With regard to the amount of change before and after administration, there was no difference between the groups when they were compared (data not shown).

### Body fat percentage

For body fat percentage, there were no changes in the NYT group or the control group (data not shown).

### Lean body mass

For lean body mass, there were no changes in the NYT group or the control group (data not shown).

### Muscle quality score

**Figure 3:**
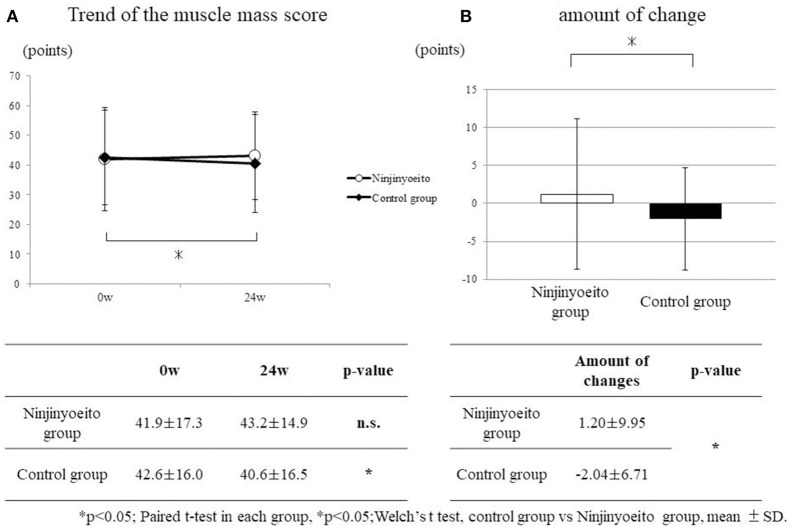
Change in the muscle quality score between both groups. The muscle quality scores of each group were compared using the paired t-test. The amount of change of each group was compared using Welch's *t*-test. Both sides were tested, and *p* < 0.05 was set as the level of significance. Ninjin'yoeito group: 61 cases; control group: 49 cases. No changes were observed in the ninjin'yoeito group, but in the control group, muscle quality scores had significantly deteriorated. With regard to the amount of change before and after administration, when both groups were compared, the ninjin'yoeito group showed significant improvement.

No changes were observed in the NYT group, but in the control group, the muscle quality score had significantly deteriorated (*p* < 0.05). With regard to the amount of change before and after administration, when the two groups were compared, a significant difference was observed (*p* < 0.05) (Figure 3).

### Estimated bone mass

For estimated bone mass, there were no changes in the NYT group or the control group (data not shown).

### Body age

There were no changes in the NYT group, but the body age had significantly deteriorated in the control group (*p* < 0.05). With regard to the amount of change before and after administration, there was no difference between the groups when they were compared (data not shown).

### Thigh circumference

For thigh circumference, there were no changes in the NYT group or the control group (data not shown).

## Discussion

Not only does frailty lead to a noticeable decline in activity of daily living (6), but it also causes an increase in the number of people requiring nursing care (7), becoming a significant burden for families and society. It should thus not be treated as a personal issue, but as a societal problem.

Frailty can be classified into three different types: physical frailty, mental and psychological frailty, and social frailty (8). Physical frailty mainly takes the form of sarcopenia. People with mental and psychological frailty display signs of depression and mild cognitive decline. Furthermore, social frailty is demonstrated by those in isolated environments, such as those who live alone or shut themselves up in their room. It is important to note that these three types of frailty are not independent of one another, as they influence each other and are reversible. Health care and nursing care professionals who often examine frail patients should help them to return to a healthy state by accurately assessing their frailty, and providing them with meal and exercise guidance, as well as medication as needed.

While routinely examining many frail patients, the author noticed that *kiketuryokyo* was at the root of the condition. So, when the patients were administered NYT, which is a typical *kiketsu* complementary agent, several of them experienced dramatic improvements in the level of frailty. Moreover, these patients' depressive symptoms also improved considerably. Therefore, these results show that NYT can be effective in treating mental and psychological frailty (5). The results of the cohort study found that the administration of NYT improved the patients' grip strength in their right and left hands, and they were able to maintain their muscle quality.

**Figure 4:**
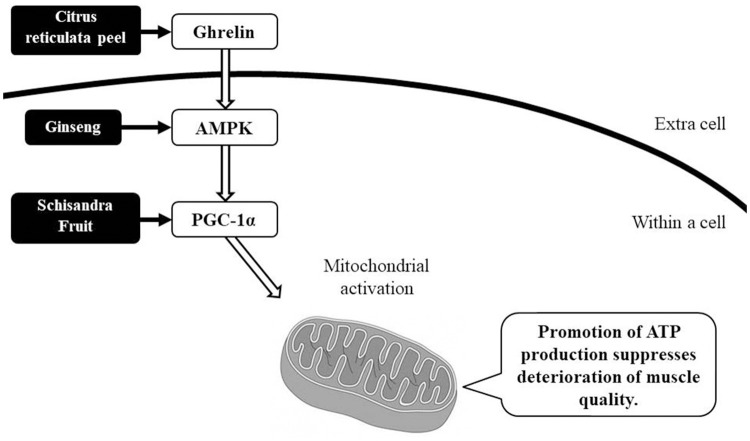
Estimated mechanism of the action of ninjin'yoeito against muscle weakness. We can surmise that citrus reticulata peel promotes ghrelin production, ginseng activates AMPK, and Schisandra fruit induces the expression of *PGC-1*α in the skeletal muscle, which activates muscle mitochondria and increases energy production efficiency.

There are many unanswered questions concerning the effect of NYT on muscles, but from the basic research reported thus far we can surmise that citrus reticulata peel promotes ghrelin production (9), ginseng activates activated protein kinase (AMPK) (10), and Schisandra fruit induces the expression of *PGC-1*α in skeletal muscle (11), which in turn activates muscle mitochondria and increases energy production efficiency (Figure 4). It is thought that NYT, which includes these crude drugs, has great potential to act as a form of “frailty treatment” in the future.

## Conclusions

This study, which included more than 100 patients, suggests that NYT improves grip strength in both hands and maintains muscle quality. Therefore, NYT, a dual deficiency of qi and blood preparation, may be effective in treating physical frailty; however, there is a need to further study the effectiveness by increasing the number of cases further.

## Data availability statement

The datasets for this manuscript are not publicly available because of patient privacy concerns but are available from the author on reasonable request.

## Author contributions

NS analyzed the data and wrote this report. KM, SS, TI, YT, HS, and TU provided the patient data.

### Conflict of interest statement

The authors declare that the research was conducted in the absence of any commercial or financial relationships that could be construed as a potential conflict of interest.
